# Impact of Subcutaneous Adipose Tissue Index Change During Neoadjuvant Chemoradiotherapy on Disease-Free Survival and Tumor Response in Patients with Locally Advanced Rectal Cancer

**DOI:** 10.7150/ijms.114915

**Published:** 2025-10-10

**Authors:** Qing Yang, Siyi Lu, Ruize Qu, Nan Zhang, Maoye Chen, Yi Zhang, Yanpeng Ma, Zhipeng Zhang, Hao Wang, Wei Fu

**Affiliations:** 1 Cancer Center, Peking University Third Hospital, Beijing, P. R. China.; 2 Beijing Key Laboratory for Interdisciplinary Research in Gastrointestinal Oncology (BLGO), Beijing, P. R. China.; 3 Department of General Surgery, Peking University Third Hospital, Beijing, P. R. China.; 4 Institute of Medical Technology, Health Science Center of Peking University, Beijing, P. R. China.; 5 Health Science Center of Peking University, Beijing, P. R. China.; 6 Department of Radiation Oncology, Peking University Third Hospital, Beijing, P. R. China.

**Keywords:** neoadjuvant chemoradiotherapy, locally advanced rectal cancer, adipose tissue, prognosis, tumor response

## Abstract

**Background & Aims:** The heterogeneity among patients with locally advanced rectal cancer (LARC) necessitates identifying predictive markers of response to neoadjuvant chemoradiotherapy (nCRT) to enable personalized treatment strategies. Adipose tissue, which reflects nutritional status and chronic inflammation, has been implicated in tumorigenesis and disease progression. This study investigated the potential of adipose tissue as a predictive marker of nCRT response and prognosis in patients with LARC.

**Methods:** We analyzed pre- and post-nCRT non-contrast computed tomography images at the third lumbar vertebral level to quantify adipose tissue in patients with LARC. We examined the relationship between changes in the subcutaneous adipose tissue index (SATI) and treatment outcomes, including disease-free survival (DFS), tumor regression grade (TRG), and tumor downstaging, using Cox proportional hazards and logistic regression analyses.

**Results:** This study included 290 patients who underwent radical surgery after nCRT. Patients with significant increases in SATI had improved DFS (*P* = 0.002) and better short-term treatment responses, including superior TRG (*P* = 0.019) and more favorable tumor downstaging (*P* = 0.005). Multivariate analyses revealed that SATI gain was an independent prognostic factor for both long-term outcomes (DFS, *P* = 0.018) and short-term treatment responses (TRG, *P* = 0.020; tumor downstaging, *P* = 0.008). Additionally, calibration and decision curve analyses demonstrated the strong predictive ability of the nomogram incorporating SATI gain for DFS.

**Conclusions:** An increase in SATI during nCRT was an independent protective factor for DFS and an independent predictor of treatment response in patients with LARC.

## 1. Introduction

Colorectal cancer (CRC) is the third most commonly diagnosed cancer and second leading cause of cancer-related mortality globally^1^. In China, rectal cancer accounts for 48.3% of all CRC cases, prompting increased oncological research on rectal cancer^2^. Many patients are initially diagnosed with locally advanced rectal cancer (LARC) because of its subtle onset. Current international guidelines recommend neoadjuvant chemoradiotherapy (nCRT) as the standard management for LARC, followed by an assessment of tumor response^3^-^5^. Patients achieving a complete clinical response (cCR) may be eligible for a "watch and wait" strategy, whereas others may require total mesorectal excision (TME). Advantages of nCRT include reduced local recurrence rates, improved resection and sphincter preservation rates, and enhanced disease-free survival (DFS).

Approximately 15-27% of patients achieve a pathological complete response (pCR), potentially avoiding TME^6^. However, 13-50% exhibit poor tumor response to nCRT, experiencing unnecessary treatment-related toxicity and side effects^7^. Thus, distinguishing patients based on their tumor responses remains a formidable challenge in the era of personalized treatment.

Recently, increasing attention has been given to the nutritional status of patients with cancer. Guidelines emphasize optimizing nutritional status because of its association with effective tumor management and improved quality of life^8^-^11^. Adipose tissue, a vital component of body composition, partially reflects nutritional status, especially in patients with cancer^11^-^13^. Moreover, adipose tissue has been implicated in the tumorigenesis and progression of various types of cancer, including CRC^14^. The mechanisms underlying the role of adipose tissue in tumorigenesis and progression are still under investigation, with current theories focusing on chronic inflammation and lipid metabolism^15^-^19^. For instance, inflammation-related mediators such as interleukin (IL)-6 and tumor necrosis factor (TNF)-α, which promote CRC development, increase during chronic inflammatory conditions. Leptin, a hormone secreted by the adipose tissue, plays a crucial role in lipid metabolism and promotes cell proliferation, migration, and invasion by activating the JAK/STAT pathway^20^. These mechanisms highlight the role of adipose tissue in tumor biology, while emphasizing that its metabolic activity can be reflected by the area occupied by adipose tissue. However, the relationship between the adipose tissue area and LARC remains unclear in clinical settings. Therefore, this study aimed to investigate whether adipose tissue area can serve as a potential marker for correlating nutritional status and chronic inflammation with therapeutic outcomes in patients with LARC.

## 2. Methods

### 2.1 Study population

We retrospectively collected data from 290 patients with LARC who underwent nCRT at Peking University Third Hospital between January 2013 and December 2022. The inclusion criteria were as follows: (1) confirmation of rectal adenocarcinoma diagnosis through pre-nCRT colonoscopy pathology, (2) diagnosis of LARC based on pre-nCRT computed tomography (CT) and magnetic resonance imaging (MRI), indicating clinical T stage 3-4 or positive clinical N stage, cT3-4 or cN+, (3) radical surgery after nCRT, and (4) availability of complete clinical data, including pre- and post-nCRT abdominopelvic CT images, inpatient records, and follow-up information.

The exclusion criteria were as follows: (1) presence of other types of malignant tumors excluding rectal cancer, (2) incomplete pre- and post-nCRT abdominal-pelvic CT images, (3) identification of metal lumbar implants on CT scans, (4) failure to undergo radical surgery after nCRT, including a “watch and wait” strategy or palliative surgery, and (5) missing follow-up data.

**Figure 1:**
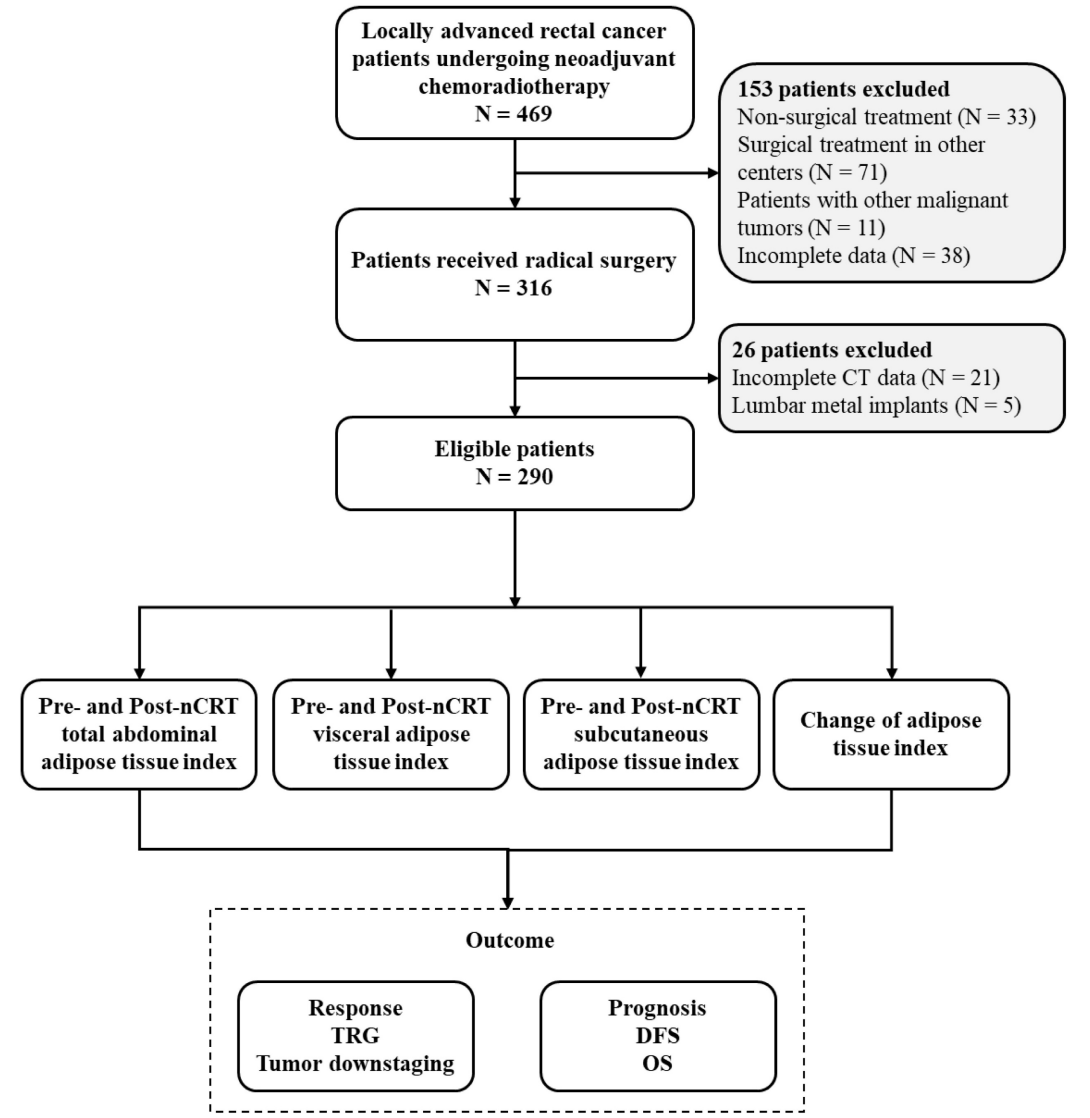
** Flow chart of the study**.

Ethical approval was obtained from the Peking University Third Hospital (IRB00006761-M2024350), and this study adhered to the tenets of the Declaration of Helsinki. The Institutional Review Board of Peking University Third Hospital waived the requirement for informed consent. A detailed flow chart of patient selection and outcomes is shown in Figure 1.

### 2.2 nCRT Treatment

All patients received the same nCRT regimen. The decision to administer nCRT or proceed with radical resection was made by a multidisciplinary team of surgeons, oncologists, pathologists, and radiologists. Radiotherapy consisted of 45-50 Gy delivered in 25 fractions according to institutional protocols. Oral capecitabine was administered at a daily dose of 1,650 mg/m² throughout radiotherapy. Pathological staging followed the American Joint Committee on Cancer (AJCC) eighth edition classification, as recommended by the National Comprehensive Cancer Network guidelines. Tumor regression grade (TRG) was categorized as follows: TRG0, absence of tumor cells; TRG1, isolated tumor cells or small clusters; TRG2, residual cancer with desmoplastic response (mild regression); and TRG3, no significant tumor cell death.

### 2.3 Clinical data

We collected clinical information on the following five aspects:

(1) Baseline data: sex, age, height, weight, and body mass index (BMI).

(2) nCRT-related indicators: pre-nCRT tumor location, size, and clinical Tumor-Node-Metastasis (TNM) stage (evaluated using CT and MRI), as well as post-nCRT hemoglobin (HGB), albumin (ALB), fibrinogen, and carcinoembryonic antigen (CEA) levels.

(3) Post-radical surgery: pathological TNM stage (ypTNM) classification according to the AJCC eighth edition standard, lymph node metastasis status, identification of tumor deposits, occurrence of lymphovascular invasion (LVI), presence of perineural invasion (PNI), pathological TRG, and tumor downstaging.

(4) Adipose tissue area was quantified using pre- and post-nCRT non-contrast abdominal-pelvic CT scans at the third lumbar vertebra.

(5) Prognostic data: overall survival rates (OS) and DFS.

### 2.4 Measurement of adipose tissue area

Non-contrast cross-sectional abdominopelvic CT scans were performed 1 week before nCRT initiation and 8-12 weeks after nCRT completion with patients in the supine position at the level of the third lumbar vertebra (L3). Adipose tissue areas were measured using ImageJ software v1.47i (National Institutes of Health, Bethesda, MD, USA), a Java-based open-source image processing software^21^,^22^. Hounsfield unit (HU) thresholds for adipose tissue were set at -190 to -30 HU. The adipose tissue encompassed the total abdominal, visceral, and subcutaneous compartments **(**Figure S1**)**. Total abdominal adipose tissue (TAT) was defined as the sum of visceral adipose tissue (VAT) and subcutaneous adipose tissue (SAT). To account for variations in patient body size, TAT, VAT, and SAT were normalized by dividing by the square of the patient's height, resulting in adjusted adipose tissue areas, recorded as the total abdominal adipose tissue index (TATI), visceral adipose tissue index (VATI), and subcutaneous adipose tissue index (SATI), respectively, in units of cm^2^/m^2^.

Patients were categorized into high and normal adipose tissue area groups based on the highest quartile^23^,^24^. The change in adipose tissue area was calculated as post-nCRT minus pre-nCRT values. Subsequently, patients were classified into fat and non-adipose tissue gain groups according to the highest quartile of this change. Quartiles were computed separately for male and female patient cohorts. For example, the highest quartile of pre-nCRT TATI was determined independently for each sex. Patients with a pre-nCRT TATI ≥ their sex-specific highest quartile were assigned to the high group.

### 2.5 Outcome parameters

The primary short-term outcomes were TRG and tumor downstaging. We categorized TRG 0-1 as a pathological good response (pGR) and TRG 2-3 as a pathological poor response (pPR). Tumor downstaging was defined as an ypTNM stage lower than the clinical TNM stage. The long-term prognostic parameters included OS and DFS, which were calculated monthly. OS was defined as the time from surgery to death, whereas DFS was defined as the period between surgery and the first tumor recurrence.

### 2.6 Statistical analysis

The normality of continuous variables was assessed using the Kolmogorov-Smirnov test. Normally distributed data are presented as mean ± standard deviation. The homogeneity of variance among groups was evaluated using Levene's test, and the independent sample t-test or Welch's t-test was used for comparative analysis. Non-normally distributed data are described using medians (interquartile ranges [IQR]), and non-parametric tests were used for group comparisons. Categorical variables were analyzed using Fisher's exact test or the chi-square test.

Univariate logistic regression analysis was performed to identify factors associated with TRG or tumor downstaging. Variables with a univariate *P*-value of less than 0.1 were included in multivariate logistic regression analysis. DFS and OS curves depicting the relationship between the non-SATI gain and SATI gain groups were constructed using the Kaplan-Meier method. Univariate Cox regression was initially conducted to examine factors related to DFS, followed by multivariate analysis, including variables with a univariate *P*-value less than 0.1. Independent prognostic factors from multivariate Cox regression analysis were incorporated into a predictive model to construct a nomogram, whose performance was validated using calibration and decision curve analysis (DCA). Statistical analyses were performed using SPSS version 26.0 (IBM Corporation, Armonk, NY, USA), with statistical significance set at *P*-value < 0.05. Additional analyses, including forest plots, Kaplan-Meier survival curves, nomograms, calibration assessments, and DCA, were conducted using R version 4.2.1.

## 3. Results

### 3.1 Patient characteristics

Based on the inclusion and exclusion criteria, 290 patients were enrolled. The cohort comprised 206 males (71%) and 84 females (29%), with a median age of 61 years (range: 22-82 years). The median follow-up duration was 34 months. Their mean BMI was 24.17 kg/m². Among the participants, 34 patients (11.7%) were classified as obese (BMI>27.9 kg/m²), 113 (39.0%) as overweight (BMI≥24.0 kg/m²), and 10 (3.4%) as underweight (BMI<18.5 kg/m²).

The tumor locations varied, with 26 patients (9%) having tumors in the upper rectum, 160 (55%) in the middle rectum, and 104 (36%) in the lower rectum. The tumor size was ≤5 cm in 173 patients (59.7%) and >5 cm in 117 (40.3%). At initial diagnosis, 234 patients (80.7%) were classified as cT2-3, while 56 (19.3%) had cT4 tumors. Additionally, 81% had suspected lymph node metastasis (cN+), whereas 19% had no lymph node involvement (cN0).

After nCRT, 48 patients (16.8%) achieved a pCR with a ypTNM stage of 0. Post-nCRT, CEA levels were within the normal range (≤5 ng/mL) in 247 patients (85.2%). The median HGB level, reflecting the patients' nutritional status, was 129 g/L (IQR 119-139), and the median ALB level was 41.8 g/L (IQR 38.8-44.4).

More than half of the patients (160, 55.2%) achieved a TRG of 0-1, indicating pGR, while only 30 patients (10.3%) had a TRG of 3. Detailed baseline characteristics of the patients are provided in Table S1.

### 3.2 Adipose tissue change during neoadjuvant therapy

We delineated three types of adipose tissue within the L3 cross-sectional area of the abdominopelvic CT scans obtained pre- and post-nCRT. Pre-nCRT measures were labeled as pre-nCRT TATI, pre-nCRT VATI, and pre-nCRT SATI, whereas post-nCRT measures were labeled as post-nCRT TATI, post-nCRT VATI, and post-nCRT SATI. Given the sex differences in the adipose distribution, we analyzed these adipose tissue types separately by sex, as detailed in Table 1.

**Figure 2:**
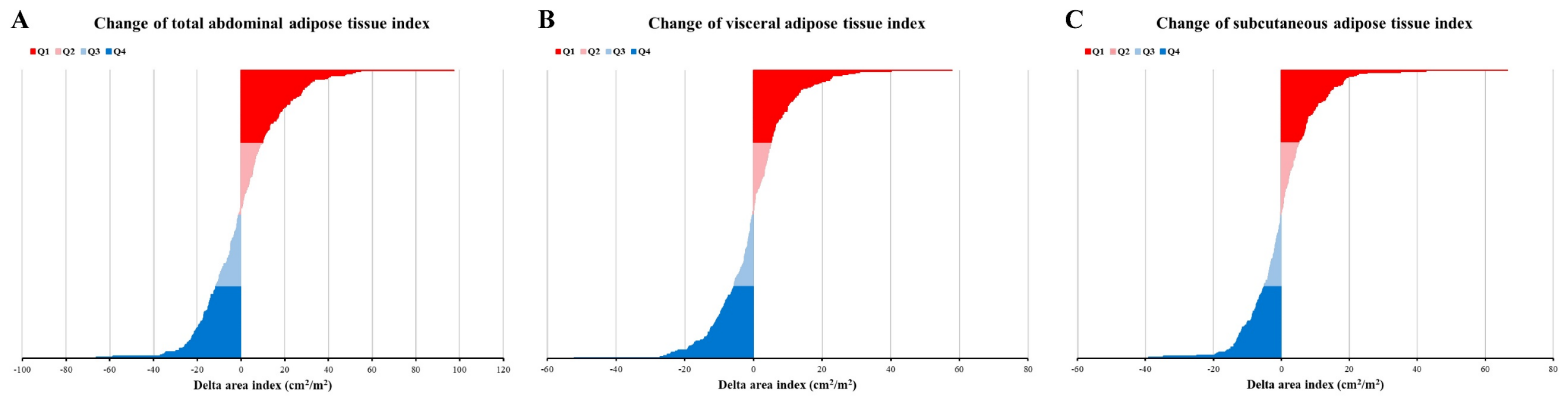
** Change of adipose tissue index.** (A) Change of total abdominal adipose tissue index; (B) Change of visceral adipose tissue index; (C) Change of subcutaneous adipose tissue index. Q1, Q2, Q3, and Q4 have represented the highest quartile, 50-75%, 25-50%, and lowest quartile, respectively.

The changes in each adipose tissue type during nCRT were assessed by subtracting pre-nCRT values from post-nCRT values, yielding changes in TATI, VATI, and SATI (cm²/m²). As depicted in Figure 2, approximately 50% of patients exhibited an increase in TATI during nCRT, while the rest demonstrated a decrease. Similar patterns were observed for VATI and SATI.

### 3.3 Prognostic impact on adipose tissue

**Figure 3:**
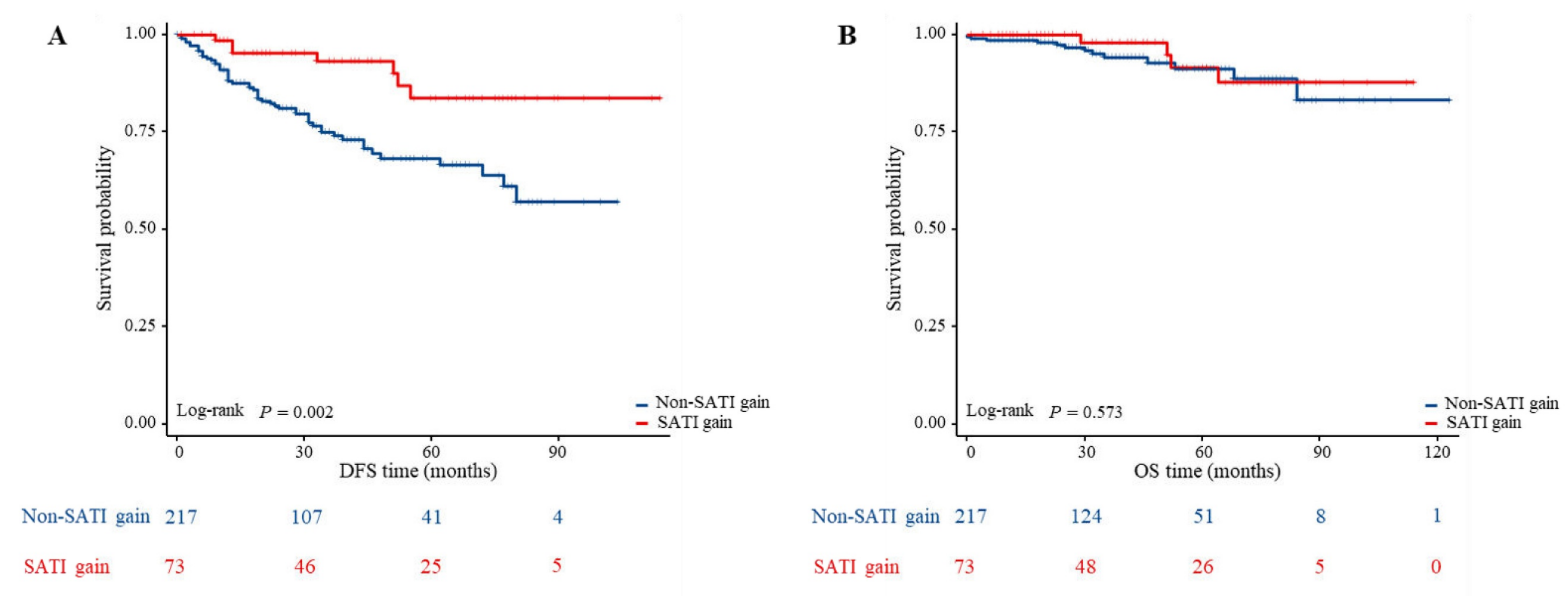
** Comparison of DFS and OS between the different changes of subcutaneous adipose tissue index in patients with LARC.** (A) Kaplan-Meier analysis for DFS rate between non-SATI gain and SATI gain groups in patients with LARC (*P* = 0.002); (B) Kaplan-Meier analysis for OS rate between non-SATI gain and SATI gain groups in patients with LARC (*P* = 0.573).

The median follow-up period was 34 months. Among the 290 patients, 61 (21.03%) experienced recurrence and 17 (5.86%) died by the last follow-up. To investigate the impact of adipose tissue on outcomes in patients with LARC undergoing nCRT, we stratified patients by adipose tissue levels and used Kaplan-Meier estimates to compare differences in DFS and OS across groups. Figure 3A shows that patients with a significant increase in SATI during nCRT (labeled as “SATI gain”) had better DFS compared to those without such an increase (labeled as “non-SATI gain”) (*P*=.002). However, OS did not differ significantly between the two groups (Figure 3B).

Conversely, no differences in DFS (Figure S2) or OS (Figure S3) were observed for other adipose tissue indicators. These findings indicate that changes in SATI were associated with DFS, whereas changes in other adipose tissue indicators were not.

### 3.4 Cox proportional regression on DFS

Based on previous findings, SATI gain was associated with improved DFS but not OS. To determine whether SATI gain is an independent protective factor for DFS, we performed a multivariate Cox regression analysis, including common factors associated with DFS such as tumor size, clinical TNM stage, ypTNM stage, lymph node metastasis, presence of tumor deposits, LVI, PNI, and CEA levels, in addition to SATI gain. As detailed in Table 2, SATI gain emerged as an independent protective factor for DFS (*P* = 0.018), with an effect comparable to the presence of tumor deposits and superior to traditional indicators such as tumor size, clinical TNM stage, lymph node metastasis, lymphovascular invasion, and neural invasion.

**Figure 4:**
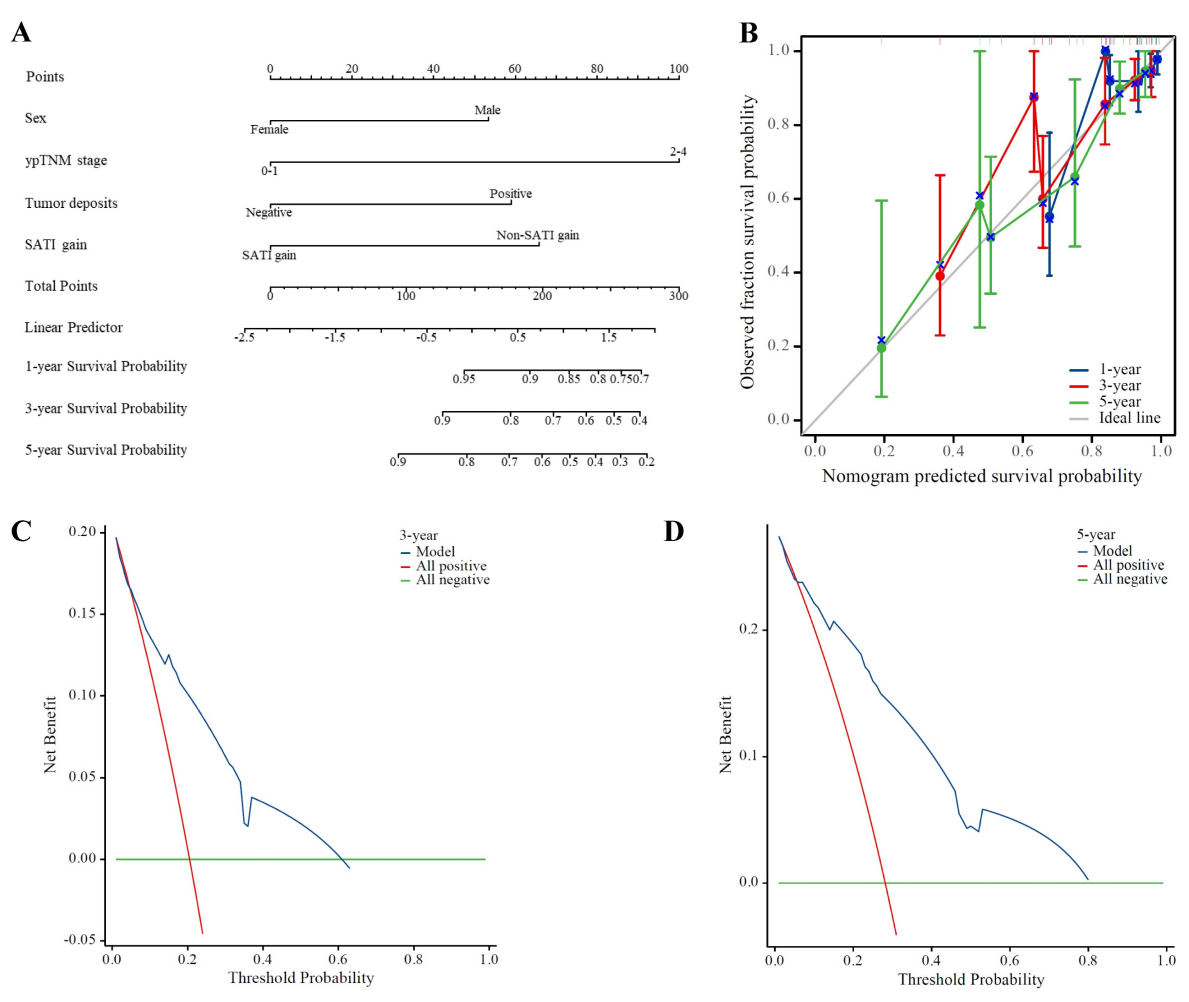
** Predictive performance of SATI gain for DFS.** (A) Nomogram; (B) Calibration curve on 1-, 3- and 5-year DFS; (C) 3-year DCA; (D) 5-year DCA. ***ypTNM*** pathological TNM stage, ***SATI*** subcutaneous adipose tissue index.

Subsequently, we incorporated significant factors from multivariate Cox regression analysis into a predictive model, including sex, ypTNM stage, presence of tumor deposits, and SATI gain. The nomogram in Figure 4A demonstrates that SATI gain has predictive power second only to ypTNM stage. The calibration curves for 1-, 3-, and 5-year DFS (Figure 4B) were progressively aligned with the reference line, indicating that our model provides increasingly accurate risk predictions over time. Additionally, DCA of the model (Figures 4C and 4D) demonstrated robust predictive performance for 3- and 5-year DFS, with particularly strong performance for 5-year DFS within the 0-80% threshold range.

Our constructed model demonstrated superior predictive performance for 3- and 5-year DFS compared to the traditional prognostic indicator, ypTNM stage.

### 3.5 Tumor response and downstaging

In addition to evaluating DFS and OS, we investigated the correlation between adipose tissue types and the short-term efficacy of nCRT, typically assessed using TRG and tumor downstaging. Univariate and multivariate logistic regression analyses were performed to examine the association between common indicators potentially related to TRG and downstaging as well as the adipose tissue indicators explored in this study.

**Figure 5:**
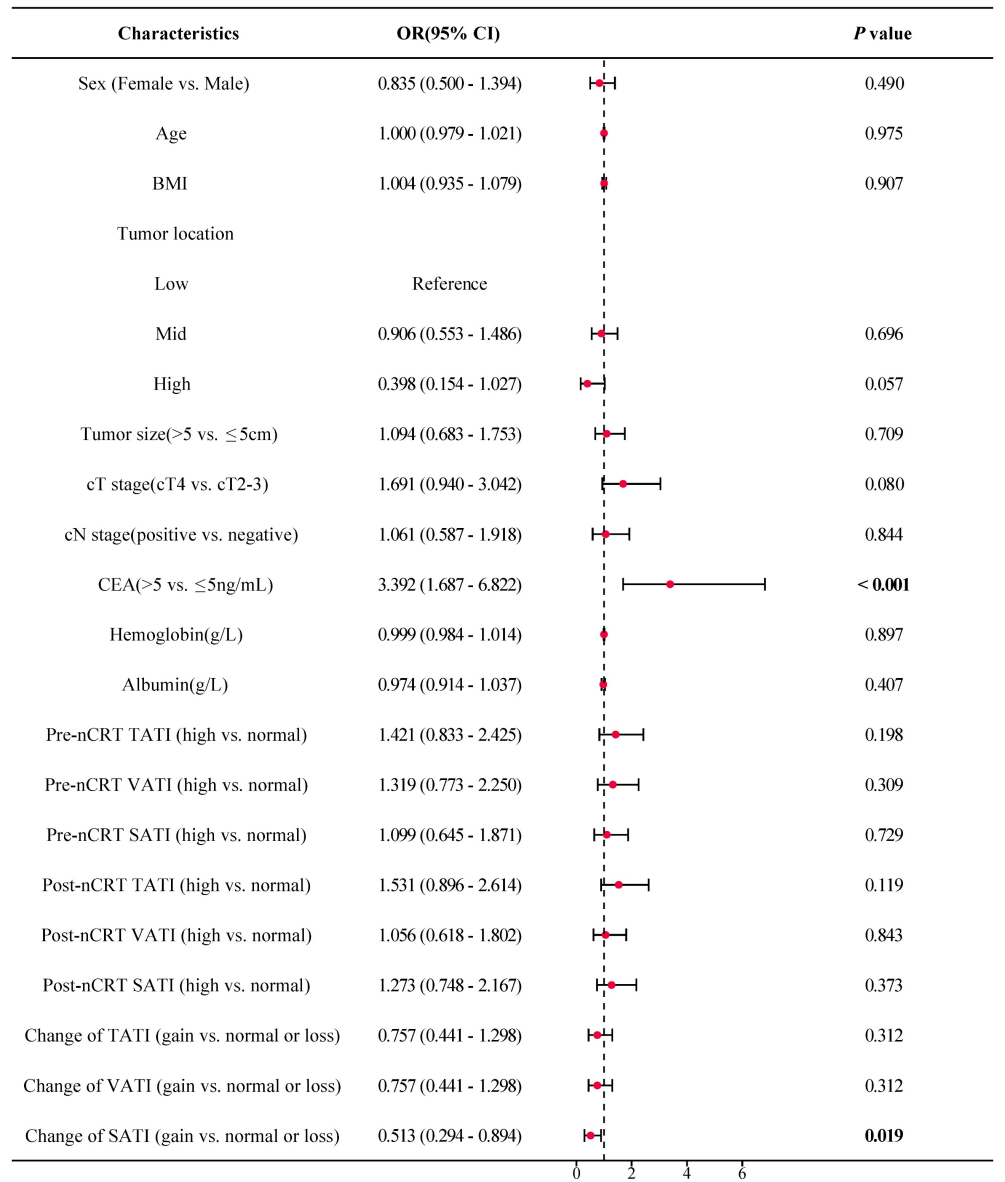
** Forest plot of TRG. *OR*** odds ratio; ***CI*** confidence interval; ***BMI*** body mass index; ***cT stage*** clinical T stage; ***cN stage*** clinical N stage; ***CEA*** carcinoembryonic antigen; ***TATI*** total abdominal adipose tissue index; ***VATI*** visceral adipose tissue index; ***SATI*** subcutaneous adipose tissue index.

**Figure 6:**
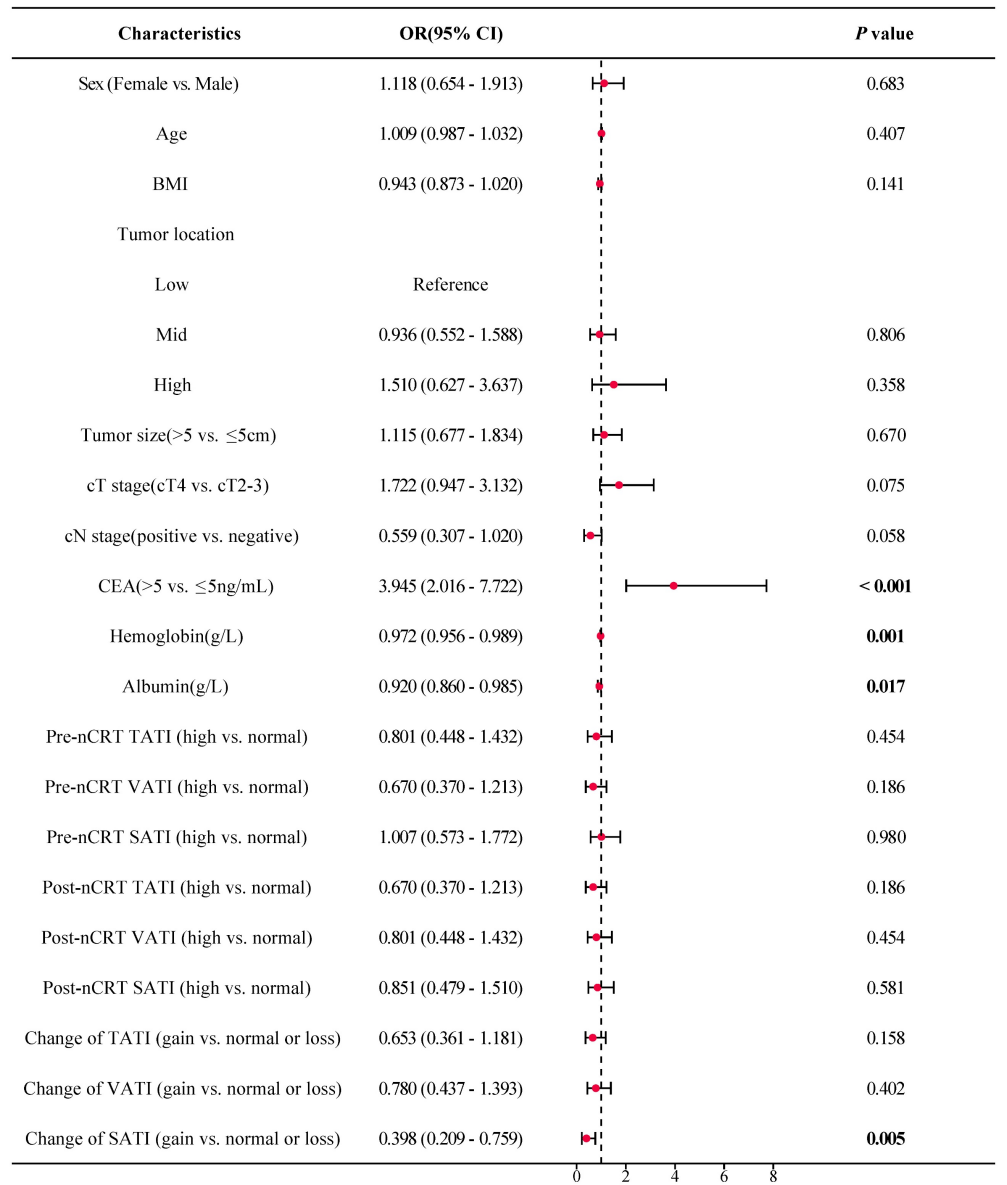
** Forest plot of tumor downstaging. *OR*** odds ratio; ***CI*** confidence interval; ***BMI*** body mass index; ***cT stage*** clinical T stage; ***cN stage*** clinical N stage; ***CEA*** carcinoembryonic antigen; ***TATI*** total abdominal adipose tissue index; ***VATI*** visceral adipose tissue index; ***SATI*** subcutaneous adipose tissue index.

As shown in Figure 5, the forest plot from the univariate logistic regression analysis revealed that SATI gain significantly differed between patients with TRG 0-1 (pGR) and those with TRG 2-3 (pPR), with SATI gain serving as a predicted factor for pGR (*P* = 0.019). Similarly, SATI gain significantly differed between the tumor downstaging and non-downstaging patient groups, acting as a protective factor for tumor downstaging (Figure 6, *P* = 0.005).

Multivariate logistic regression analysis further supported that SATI gain was an independent protective factor for both pGR (Table 3) and tumor downstaging (Table 4), with *P*-values of 0.020 and 0.008, respectively. SATI gain demonstrated superior predictive capability compared to clinical T stage, N stage, and tumor location.

## 4. Discussion

Identifying markers to distinguish patients who benefit from nCRT has long been a focus because of the heterogeneity among patients with LARC; however, accurate, rapid, and cost-effective methods remain unclear in clinical practice. In this retrospective study, potential markers were identified to predict the response to nCRT treatment (short-term outcomes) and prognosis (long-term outcomes) by measuring the adipose tissue index at the L3 level on pre- and post-nCRT CT scans. SATI was associated with DFS. First, an increase in SATI during nCRT correlated with better DFS and served as an independent protective factor. Second, increased SATI was associated with improved short-term outcomes, including a higher proportion of pGR (TRG 0-1) and tumor downstaging. Third, increased SATI independently predicted favorable short-term outcomes of nCRT. These findings demonstrated that increased SATI during nCRT could serve as a useful marker for identifying patients who will benefit from this treatment modality. Additionally, changes in the adipose tissue index may reflect the nutritional status of patients with LARC and correlate with treatment efficacy and prognosis.

The nutritional status of patients with cancer is increasingly recognized as a factor contributing to better patient outcomes^8^. However, debate persists regarding the use of specific body composition measures, such as VAT and SAT, as indicators of cancer outcomes. A retrospective study on gastric cancer classified patients by obesity status and the VAT-to-SAT ratio, revealing that patients who are not overweight or obese with a high VAT/SAT ratio had worse prognosis (HR, 1.89, 95% CI: 1.28-2.77)^25^. Similar conclusion was observed in CRC through a multicenter randomized controlled trial, which demonstrated an association between high VAT-to-TAT ratio and increased rates of cancer recurrence (HR, 5.78, 95% CI: 3.66-7.95, *P* = 0.02) and mortality (HR, 5.92, 95% CI: 4.04-8.00, *P* = 0.02)^26^. Another study in this field reported that lower VAT mass was associated with worse outcomes in various cancer types, including CRC^27^. In summary, although the impact of VAT on gastrointestinal cancer remains uncertain, emerging evidence suggests that higher VAT may be associated with poorer prognoses.

Findings from studies on SAT differ from those on VAT. In a retrospective study of 158 patients with advanced gastric cancer treated with dual PD-1 and HER2 blockade, a higher SATI level was found to be an independent protective factor for progression-free survival (HR, 0.628, 95% CI: 0.410-0.962, *P* = 0.032) and was associated with a better treatment response than a lower SATI level (62.6% vs. 34.3%, *P* = 0.004)^28^. Another retrospective study of 987 patients with CRC in AJCC stages I-III demonstrated that a higher preoperative SATI independently predicted longer DFS (HR, 0.505; 95% CI: 0.266-0.957, *P* = 0.036)^29^. Regarding short-term outcomes, SAT was associated with improved locoregional control following radiotherapy in a large-scale study involving 1,957 patients with head and neck cancer, supporting the view that higher SAT levels may correlate with increased radiosensitivity^30^. However, similar large-cohort evidence is currently lacking for rectal cancer. Overall, previous studies have indicated that higher SAT is associated with favorable outcomes; however, consistent identification of detailed outcome measures across these studies is lacking.

In our study, increased SATI levels served as an independent protective factor for both short- and long-term outcomes in patients with LARC. Neither high SATI pre- nor post-nCRT correlated with DFS or OS (**Figures S2-3**); however, changes in SATI during nCRT showed a stronger correlation with DFS and reflected the tumor response to nCRT, which differs from previously reported findings^28^. This novel parameter may accurately reflect dynamic changes in nutritional status and predict short- and long-term outcomes in patients with LARC.

Improving the curative efficacy of nCRT in LARC remains a key challenge. Several novel approaches, including modifying radiation dose, adjusting chemotherapy drugs, combining nCRT with immunotherapy or targeted therapy, and optimizing treatment duration, have demonstrated promising effects to some extent^31^-^33^. However, further high-quality studies are needed to explore new strategies that improve efficacy and prognosis without increasing side effects or long-term complications^33^,^34^. Based on our findings, we hypothesize that enhancing patients' nutritional status (particularly by increasing SAT) during nCRT may augment the efficacy of nCRT for LARC.

The association between SATI gain and improved short- and long-term outcomes may be explained by the following hypotheses: adipose tissue content and distribution reflect lipid metabolism. Increased SATI may exert a protective effect on treatment efficacy and prognosis through specific lipid metabolism pathways that influence tumor progression, resulting in different treatment responses and prognoses among patients with LARC. Nutritional and metabolic status can be substantial affected in patients with cancer, with high metabolic decomposition to support tumor cells, thereby consuming adipose tissue^11^-^13^. Moreover, adipose tissue exerts distinct effects at different locations. SAT exhibits greater metabolic stability and resistance to lipolysis than VAT^35^,^36^. From the perspective of the energy storage function of the SAT, increased SAT in these patients may reflect better resistance to the negative consumptive effects of tumors, resulting in a better response to nCRT and improved DFS.

Adiponectin, a major hormone predominantly secreted by the SAT, has been demonstrated to exert antitumor effects in vivo by downregulating angiogenesis^37^,^38^. It inhibits the mTOR pathway through AMPK activation, thereby suppressing cell proliferation and growth^39^. Additionally, under high-fat diet conditions, which pose a risk factor for CRC, adiponectin can inhibit the proliferation of colonic epithelial cells by suppressing the mTOR pathway^40^. This finding suggests that adiponectin may help prevent CRC, particularly in patients with increased SATI levels, potentially reducing nCRT resistance and tumor recurrence.

In addition to SATI gain, female sex was identified as an independent protective factor for DFS. This phenomenon can be attributed to differences in adipose tissue distribution between males and females. Males tend to accumulate more adipose in the abdominal cavity, specifically VAT, while females accumulate more SAT, particularly in the buttocks and thighs^41^-^43^. To account for these disparities in adipose tissue types and distribution between sexes, all quartiles of adipose tissue indices were calculated separately for male and female patients.

However, our study has some limitations. First, the single-center retrospective study design introduces inevitable selection bias. Second, the lower proportion of females in our cohort (84 of 290 patients) may have led to an overestimation of the effect of sex on DFS. Third, owing to data availability constraints, only two time points were included in the analysis, without considering the postoperative time point. Fourth, surgical complications were not included because of data availability; however, these complications could potentially affect long-term outcomes.

To the best of our knowledge, this study represents the largest analysis of the dynamic changes of adipose tissue index during nCRT and their relationship with tumor response and survival in a homogeneous group of patients with LARC. A better understanding of CT-based adipose tissue measurements may play a crucial role in optimizing patient conditions and enabling more accurate pre-nCRT risk stratification.

## 5. Conclusion

Changes in SATI, based on non-contrast CT, during nCRT can predict short-term treatment response and DFS in patients with LARC and may serve as a potential predictive marker for nCRT efficacy and prognosis.

## Supplementary Material

Supplementary figures and table.

## Figures and Tables

**Table 1:** Adipose tissue index in different sex patients.

Adipose tissue index (cm^2^/m^2^)	Male N = 206	Female N = 84	*P* value
Pre-nCRT TATI, mean ± sd	96.066 ± 42.484	115.030 ± 39.837	**< 0.001**
Pre-nCRT VATI, median (IQR)	53.072 (32.135, 73.318)	49.626 (33.024, 62.176)	0.125
Pre-nCRT SATI, median (IQR)	40.102 (30.499, 50.745)	62.763 (51.944, 79.047)	**< 0.001**
Post-nCRT TATI, mean ± sd	97.828 ± 39.842	111.800 ± 39.401	**0.007**
Post-nCRT VATI, median (IQR)	55.242 (35.877, 71.931)	43.525 (32.689, 63.401)	**0.009**
Post-nCRT SATI, median (IQR)	40.106 (31.178, 52.006)	63.729 (51.902, 79.815)	**< 0.001**

***TATI*** total abdominal adipose tissue index; ***VATI*** visceral adipose tissue index; ***SATI*** subcutaneous adipose tissue index

**Table 2:** Multivariate Cox proportional hazard model of DFS

Characteristics	Total(N)		Multivariate analysis	
			Hazard ratio (95% CI)	*P* value
Sex	290			
Male	206		Reference	
Female	84		0.386 (0.193 - 0.771)	**0.007**
Tumor size	290			
≤5cm	173		Reference	
>5cm	117		1.130 (0.629 - 2.029)	0.682
cTNM stage	290			
Stage II	53		Reference	
Stage III-IV	237		2.284 (0.864 - 6.038)	0.096
ypTNM stage	290			
Stage 0-I	142		Reference	
Stage II-IV	148		2.973 (1.313 - 6.730)	**0.009**
Lymph nodes metastasis	290			
Negative	243		Reference	
Positive	47		1.139 (0.598 - 2.171)	0.692
Tumor deposits	290			
Negative	240		Reference	
Positive	50		2.154 (1.139 - 4.072)	**0.018**
LVI	290			
Negative	272		Reference	
Positive	18		0.811 (0.329 - 1.996)	0.648
PNI	290			
Negative	253		Reference	
Positive	37		1.862 (0.971 - 3.573)	0.061
CEA	290			
≤5 ng/mL	246		Reference	
>5 ng/mL	44		0.984 (0.521 - 1.858)	0.961
Hemoglobin(g/L)	290		0.991 (0.972 - 1.011)	0.383
Fibrinogen(g/L)	290		1.418 (0.926 - 2.172)	0.108
Albumin(g/L)	290		0.966 (0.883 - 1.056)	0.445
Change of SATI	290			
Normal or loss	217		Reference	
Gain	73		0.373 (0.165 - 0.841)	**0.018**

***CI*** confidence interval, ***cTNM*** clinical TNM stage, ***ypTNM*** pathological TNM stage, ***CEA*** carcinoembryonic, ***LVI*** lymphovascular invasion, ***PNI*** perineural invasion, ***SATI*** subcutaneous adipose tissue index.

**Table 3:** Multivariate logistic regression analysis of TRG

Characteristics	Total(N)		Multivariate analysis	
			Odds Ratio (95% CI)	*P* value
Tumor location	290			
Low	104		Reference	
Mid	160		0.924 (0.552 - 1.548)	0.765
High	26		0.344 (0.128 - 0.921)	**0.034**
cT stage	290			
cT2-3	234		Reference	
cT4	56		1.815 (0.974 - 3.383)	0.061
CEA	290			
≤5 ng/mL	247		Reference	
>5 ng/mL	43		3.206 (1.560 - 6.588)	**0.002**
Change of SATI	290			
Normal or loss	217		Reference	
Gain	73		0.506 (0.285 - 0.899)	**0.020**

***cT stage*** clinical T stage; ***CEA*** carcinoembryonic antigen; ***SATI*** subcutaneous adipose tissue index.

**Table 4:** Multivariate logistic regression analysis of tumor downstaging

Characteristics	Total(N)		Multivariate analysis	
			Odds Ratio (95% CI)	*P* value
cT stage	290			
cT2-3	234		Reference	
cT4	56		1.656 (0.851 - 3.223)	0.138
cN stage	290			
Negative	55		Reference	
Positive	235		0.549 (0.286 - 1.052)	0.071
CEA	290			
≤5 ng/mL	247		Reference	
>5 ng/mL	43		3.564 (1.763 - 7.201)	**< 0.001**
Hemoglobin (g/L)	290		0.976 (0.956 - 0.996)	**0.019**
Albumin (g/L)	290		0.989 (0.909 - 1.075)	0.788
Change of SATI	290			
Normal or loss	217		Reference	
Gain	73		0.401 (0.203 - 0.790)	**0.008**

***cT stage*** clinical T stage; ***cN stage*** clinical N stage; ***CEA*** carcinoembryonic antigen; ***SATI*** subcutaneous adipose tissue index.

## Abbreviations

AJCC: American Joint Committee on Cancer
ALB: albumin
BMI: body mass index
CEA: carcinoembryonic antigen
CI: confidence interval
CRC: colorectal cancer
CT: computed tomography
DCA: decision curve analysis
DFS: disease-free survival
HGB: hemoglobin
HR: hazard ratio
IL: interleukin
IQR: interquartile range
LARC: locally advanced rectal cancer
LVI: lymphovascular invasion
MRI: magnetic resonance imaging
nCRT: neoadjuvant chemoradiotherapy
NCCN: National Comprehensive Cancer Network
OS: overall survival
pCR: pathological complete response
PD-1: programmed death-1
pGR: pathological good response
PNI: perineural invasion
pPR: pathological poor response
SAT: subcutaneous adipose tissue
SATI: subcutaneous adipose tissue index
TAT: total abdominal adipose tissue
TATI: total abdominal adipose tissue index
TME: total mesorectal excision
TNF: tumor necrosis factor
TNM: tumor-node-metastasis
TRG: tumor regression grade
VAT: visceral adipose tissue
VATI: visceral adipose tissue index
