# Supplementary material for: Impact of Subcutaneous Adipose Tissue Index Change During Neoadjuvant Chemoradiotherapy on Disease-Free Survival and Tumor Response in Patients with Locally Advanced Rectal Cancer
Source: Int J Med Sci. 2025 Oct 10;22(16):4313–24. doi: 10.7150/ijms.114915 (PMC12595343; doi:10.7150/ijms.114915)
Supplement: Supplementary file 1 — Supplementary figures and table. [file ijmsv22p4313s1.pdf]

Impact of Subcutaneous Adipose Tissue Index Change During Neoadjuvant Chemoradiotherapy on Disease-Free Survival and Tumor Response in Patients With Locally Advanced Rectal Cancer

Qing Yang[1, 2, 3 #], Siyi Lu[1, 2, 3 #], Ruize Qu[1, 2, 3 #], Nan Zhang[4], Maoye Chen[5], Yi Zhang[1, 2, 3], Yanpeng Ma[1, 2, 3], Zhipeng Zhang[1, 2, 3 *], Hao Wang[1, 6 *], and Wei Fu[1, 2, 3 *]

[1]Cancer Center, Peking University Third Hospital, Beijing, P. R. China

[2]Beijing Key Laboratory for Interdisciplinary Research in Gastrointestinal Oncology (BLGO), Beijing, P. R. China

[3]Department of General Surgery, Peking University Third Hospital, Beijing, P. R. China

[4]Institute of Medical Technology, Health Science Center of Peking University, Beijing, P. R. China

[5]Health Science Center of Peking University, Beijing, P. R. China

[6]Department of Radiation Oncology, Peking University Third Hospital, Beijing, P. R. China

[#]These authors contributed equally to the work

[*]**Corresponding authors**

Zhipeng Zhang, Department of General Surgery, Cancer Center, Peking University Third Hospital, 49 North Garden Rd., Haidian District, Beijing 100191, P.R. China.

E-mail: zhangzhipeng06@126.com

Hao Wang, Department of Radiation Oncology, Cancer Center, Peking University Third Hospital, 49 North Garden Rd., Haidian District, Beijing 100191, P.R. China.

E-mail: wanghaobysy@bjmu.edu.cn

Wei Fu, MD, Department of General Surgery, Peking University Third Hospital, 49 North

Garden Rd., Haidian District, Beijing 100191, P.R. China. E-mail: fuwei@bjmu.edu.cn.

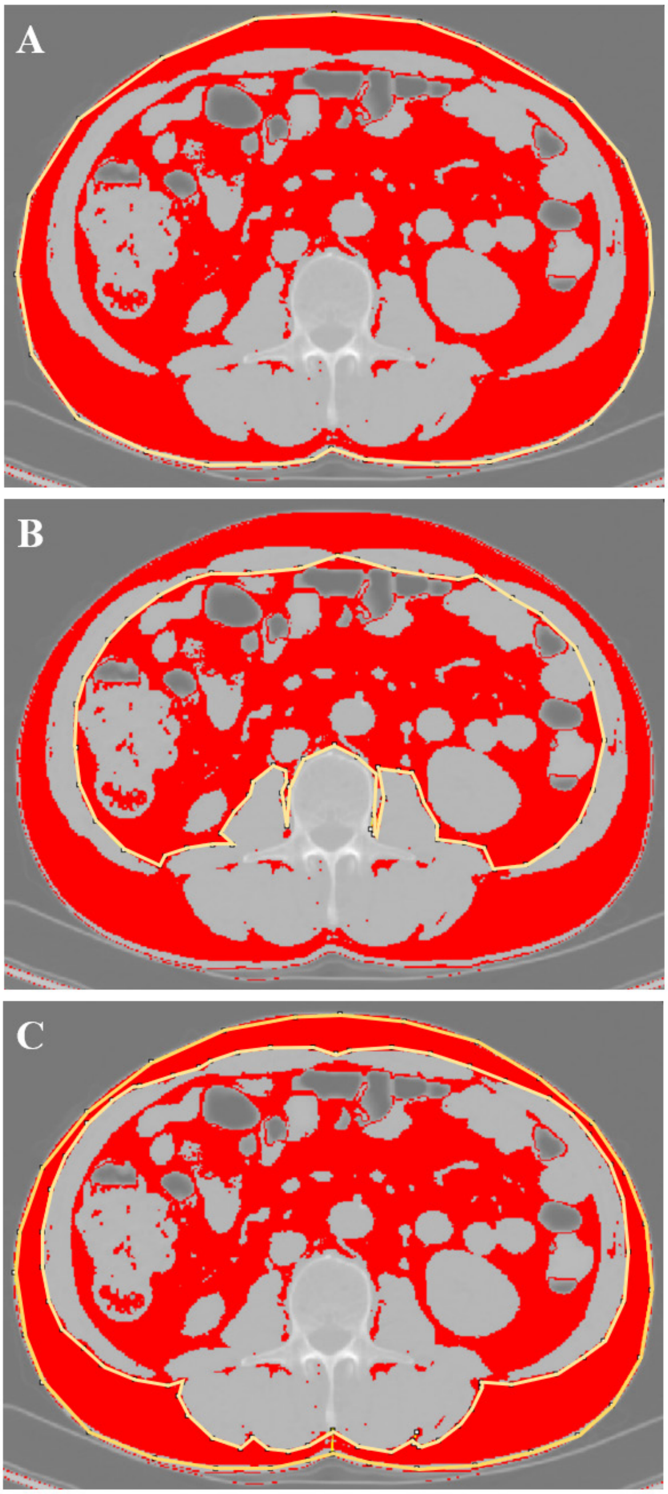

Figure S1. CT-based cross-sectional image at the third lumbar (L3) measured for TATA (A), VATA (B), and SATA (C) using ImageJ software v1.47i (National Institutes of Health, Bethesda, MD). The area delineated by the yellow solid line represents the region of interest.

Table S1. Baseline characteristics of the patients

Characteristics	Overall
**Sex, n (%)**	
Male	206 (71%)
Female	84 (29%)
**Age, median (IQR)**	61 (53, 68)
**BMI, mean ± sd**	24.167 ± 3.2384
**Tumor location, n (%)**	
High	26 (9%)
Mid	160 (55.2%)
Low	104 (35.9%)
**Tumor size, n (%)**	
≤5cm	173 (59.7%)
>5cm	117 (40.3%)
**Clinical T stage, n (%)**	
2-3	234 (80.7%)
4	56 (19.3%)
**Clinical N status, n (%)**	
Negative	55 (19%)
Positive	235 (81%)
**ypTNM, n (%)**	
0	48 (16.6%)
I-IV	242 (83.4%)
**CEA, n (%)**	
≤5 ng/L	247 (85.2%)
>5 ng/L	43 (14.8%)
**Hemoglobin, median (IQR)**	129 (119, 139)
**Albumin, median (IQR)**	41.8 (38.8, 44.375)
**TRG, n (%)**	
0	50 (17.2%)
1	110 (37.9%)
2	100 (34.5%)
3	30 (10.3%)

BMI body mass index, ***ypTNM*** pathological TNM stage, ***CEA*** carcinoembryonic antigen, ***TRG***

tumor regression grade

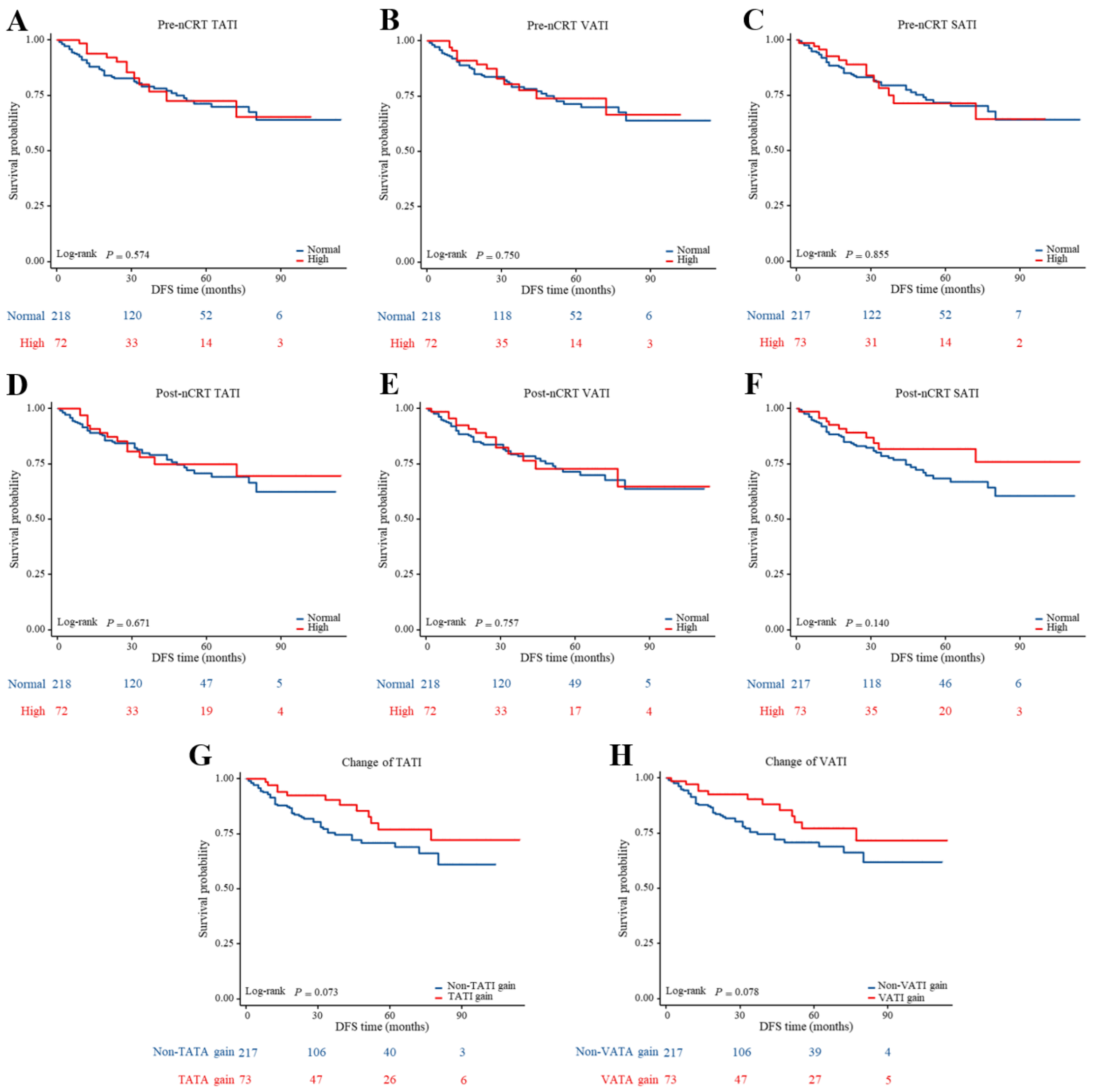

Figure S2. Kaplan-Meier analysis for DFS according to adipose tissue area and change of adipose tissue index.

(A) Kaplan–Meier analysis for DFS rate between normal pre-TATI and high pre-TATI groups in LARC patients ($P = 0.574$); (B) Kaplan–Meier analysis for DFS rate between normal pre-VATI and high pre-VATI groups in LARC patients ($P = 0.750$); (C) Kaplan–Meier analysis for DFS rate between normal pre-SATI and high pre-SATI groups in LARC patients ($P = 0.855$); (D) Kaplan–Meier analysis for DFS rate between normal post-TATI and high post-TATI groups in LARC patients ($P = 0.671$); (E) Kaplan–Meier analysis for DFS rate between normal post-VATI and high post-VATI groups in LARC patients ($P = 0.757$); (F) Kaplan–Meier analysis for DFS rate between

normal post-SATI and high post-SATI groups in LARC patients (*P* = 0.140); (G) Kaplan–Meier analysis for DFS rate between non-TATI gain and TATI gain groups in LARC patients (*P* = 0.073); (H) Kaplan–Meier analysis for DFS rate between non-VATI gain and VATI gain groups in LARC patients (*P* = 0.078).

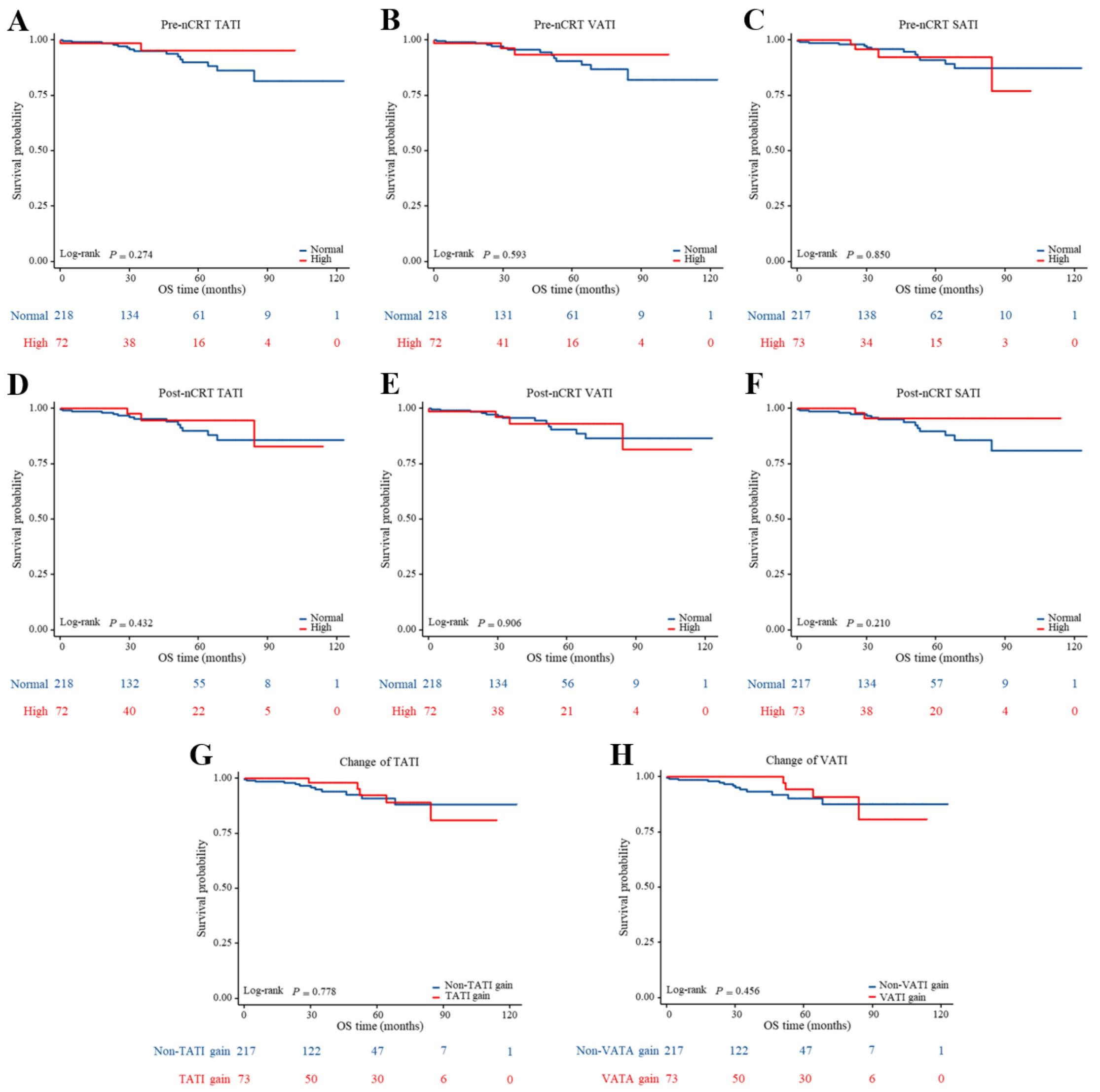

Figure S3. Kaplan-Meier analysis for OS according to adipose tissue area and change of adipose tissue area.

(A) Kaplan–Meier analysis for OS rate between normal pre-TATA and high pre-TATA groups in LARC patients (*P* = 0.274); (B) Kaplan–Meier analysis for OS rate between normal pre-VATA and high pre-VATA groups in LARC patients (*P* = 0.593); (C) Kaplan–Meier analysis for OS rate between normal pre-SATA and high pre-SATA groups in LARC patients (*P* = 0.850); (D) Kaplan–Meier analysis for OS rate between normal post-TATA and high post-TATA groups in LARC patients (*P* = 0.432); (E) Kaplan–Meier analysis for OS rate between normal post-VATA and high post-VATA groups in LARC patients (*P* = 0.906); (F) Kaplan–Meier analysis for OS rate between normal post-

SATA and high post-SATA groups in LARC patients (*P* = 0.210); (G) Kaplan–Meier analysis for OS rate between non-TATA gain and TATA gain groups in LARC patients (*P* = 0.778); (H) Kaplan–Meier analysis for OS rate between non-VATA gain and VATA gain groups in LARC patients (*P* = 0.456).
